# Are industry-funded charities promoting “advocacy-led studies” or “evidence-based science”?: a case study of the International Life Sciences Institute

**DOI:** 10.1186/s12992-019-0478-6

**Published:** 2019-06-03

**Authors:** Sarah Steele, Gary Ruskin, Lejla Sarcevic, Martin McKee, David Stuckler

**Affiliations:** 1 Department of Politics and International Studies, University of Cambridge, Cambridge, UK; 2 Jesus College, Jesus Lane, Cambridge, CB58BL UK; 3 U.S. Right to Know, Cambridge, USA; 4 Department of Public Health and Policy, London School of Hygiene & Tropical Medicine, London, UK; 5 Dondena Research Centre and Department of Policy Analysis and Public Management, University of Bocconi, Milan, Italy

**Keywords:** International Life Sciences Institute, Industry funding, Lobbying, Advocacy, Conflicts of interest

## Abstract

**Background:**

Industry sponsorship of public health research has received increasing scrutiny, and, as a result, many multinational corporations (MNCs), such as The Coca-Cola Company and Mars Inc., have committed to transparency with regard to what they fund, and the findings of funded research. However, these MNCs often fund charities, both national and international, which then support research and promote industry-favourable policy positions to leaders. We explore whether one industry funded charity, the International Life Sciences Institute (‘ILSI’), is the scientifically objective, non-lobby, internationally-credible body that it suggests it is, so as to aid the international health and scientific communities to judge ILSI’s outputs.

**Methods:**

Between June 2015 and February 2018, U.S. Right to Know), a non-profit consumer and public health group, submitted five U.S. state Freedom of Information requests (FOIs) to explore ILSI engagement with industry, policy makers, and/or researchers, which garnered a total of 17,163 pages for analysis. Two researchers explored these documents to assess the activities and conduct of ILSI against its purported objectives.

**Results:**

Within the received documents we identified instances of ILSI seeking to influence research, conferences, public messages, and policy, including instances of punishments for ILSI bodies failing to promote industry-favourable messaging. We identified ILSI promoting its agenda with national and international bodies to influence policy and law, causing the World Health Organization to withdraw from official relations with what it now considers a private sector entity.

**Conclusions:**

ILSI seeks to influence individuals, positions, and policy, both nationally and internationally, and its corporate members deploy it as a tool to promote their interests globally. Our analysis of ILSI serves as a caution to those involved in global health governance to be wary of putatively independent research groups, and to practice due diligence before relying upon their funded studies and/or engaging in relationship with such groups.

## Background

Against a backdrop of growing consumer mistrust and calls for greater openness, an emerging group of multinational corporations (‘MNCs’) in the food, beverage and supplement industries published lists of their partnerships and sponsored research as part of a commitment to greater transparency [1]. These lists detail extensive collaborations and research funding, including the payment of contributions and membership dues to professional, public, and charitable organisations dedicated to promoting science and public health. The Coca-Cola Company (‘Coke’), for example, makes membership payments to: the American Society for Nutrition; the Calorie Control Council; various constituents of the International Life Sciences Institute (‘ILSI’); the International Food Information Council (‘IFIC’); the International Stevia Council; the Food Science Strategic Policy Alliance; the National Academies of Sciences, Engineering & Medicine; the Food Forum; the Juice Products Association; Purdue University; the IOM Food Forum; the Healthy Weight Commitment Foundation; the Industry Nutrition Advisory Panel of the American Heart Association; the Preventative Cardiovascular Nurses Association; the Center for Food Integrity; the International Scientific Association of Probiotics and Prebiotics; and the Friends of Winship [2]. While some of these organisations have mechanisms to maintain their independence, some have been accused of acting as “front groups”, pushing industry positions covertly to the public, policy makers and professionals.

For instance, Mars Inc’s Vice President of Public Affairs, Matthias Berninger, declared that his MNC (which sells highly processed foods and beverages, amongst other products, worldwide), would withdraw from bodies that promote industry positions by stealth, including ILSI, due to its involvement “in advocacy-led studies” [1]. This corporate statement supported a long-held belief by many industry watchdog organisations that ILSI serves as one of many pseudo-scientific front-groups, pushing industry-favourable positions to policy makers and international bodies under the guise of neutral scientific endeavour [3, 4]. Other organisations that have been criticised for working internationally to promote industry favourable positions by stealth include the Global Energy Balance Network (GEBN), which has since closed, and IFIC, a science communications charity, both of which worked with ILSI [5].

ILSI is comprised of 18 bodies, each of which cover specific topics or geographical regions, promoting “global partnerships for a healthier world”. These include: the ILSI Research Foundation, ILSI Health and Environmental Institute, ILSI Europe, ILSI North America, ILSI Mexico, ILSI Mesoamerica, ILSI North Andean, ILSI Brazil, ILSI Argentina, ILSI South Andean, ILSI Middle East, ILSI South Africa, ILSI Southeast Asia Region, ILSI Taiwan, ILSI Korea, ILSI India, ILSI Focal Point in China, and ILSI Japan [6]. Global in nature, ILSI clearly states that it is transparent, and that none of its bodies “lobby, conduct lobbying activities, or make policy recommendations” [7]. Indeed, ILSI has long maintained its independence and scientific rigor, asserting that, through the exchange of ideas and information between scientists, academics, government, industry, and the private sector, [8] it acts as an objective body that provides “science that improves human health and well-being and safeguards the environment” [9]. Suzanne Harris, then executive director of ILSI, stated that ILSI has no “hidden agenda” and that its “work is balanced and directed toward the public good rather than the commercial interests of its corporate members”, and is not a “lobby group”, but is a public charity exempt from taxation under US Internal Revenue Code § 501(c)(3) and, as such, has refrained from lobbying [8]. Harris highlighted ILSI’s dedication to “the application of evidence-based science”, [8] a position it maintains in the face of Mars Inc’s withdrawal.

With ILSI funded research appearing in many medical, public health and nutrition, and general science journals, we believe it is critical for our colleagues to be able to rigorously assess its outputs and funded research, and whether it is engaged in lobbying and advocacy internationally. While industry collaborations with academic partners can be fruitful and produce valuable research, industry funding through covert mechanisms using third parties that obscure the funding sources can make appraisal of conflicts of interest (‘COIs’) difficult and can thwart effective evidence-based public health decision-making [10, 11]. We therefore use ILSI as a case study—a lens—through which one can explore the larger global issue of putatively independent, but heavily industry funded or influenced bodies that can affect research and national and global level policy deliberations. We draw on documents obtained using Freedom-of-Information requests (FOIs) to ask if ILSI acts, as it states, to promote the public interest and health, or if it appears to be trying to influence global health policies covertly on behalf of industry. Specifically, we question: Is ILSI engaged in advocacy for industry positions, and lobbying national and international bodies?

## Methods

Between June 2015 and October 2017, U.S. Right to Know (‘USRTK’), a non-profit consumer and public health group, submitted five U.S. state Freedom of Information requests (hereafter, FOIs) to reveal the scale and nature of engagement with industry [12]. These FOIs were sent to individuals who were selected because they had openly received funding from MNCs in the food and beverage industries and because they were in positions or institutions covered by the relevant legislation. The requests sought emails and attached documents from these individuals, with the aim of exploring their relationships with industry, and between industry and public institutions like the Centre for Disease Control (‘CDC’), charities like ILSI, policy makers, and other collaborating scholars (including scientists and academics).

Within the 17,163 pages returned by these requests, USRTK identified extensive information on ILSI, and thereby provided the batch to a researcher (SS) who independently read and explored the relationship ILSI has to industry. Notably, two researchers (GR and SS) had closely read all the documents to identify whether ILSI is engaged in lobbying, advocacy, or promoting the public good. Due to the way in which the documents were delivered—imaged PDFs in multiple files—the two researchers undertook manual reading.

The first researcher (GR) close read the documents for instances where ILSI’s conduct and motivation were detailed and then extracted relevant emails or attachments. This manual reading was repeated independently by the second researcher at a different institution (SS). The researchers then compared and discussed the findings and analysed the documents for instances where ILSI was confronted with issues of transparency, objectivity, lobbying opportunities, or engagement with international bodies, including the WHO. We did not seek to create a statistical sample, but rather to qualitatively explore ILSI’s conduct. Where there was disagreement about the documents and/or an interpretation, a full group meeting was convened, and the documents discussed.

Because of the imperfect nature of an FOI request, we may not have received all documents, and as such we do not claim the sample is comprehensive. We present the FOIs “in their own words” to allow the reader to make their own judgements. We have acted to reduce bias by reporting all emails directly referenced in an online database with weblinks, so that our interpretations are accessible to all readers, and to provide the full PDF batches received for readers and other researchers to consult. As is best practice in qualitative research, within the emails we received, we sought actively to identify instances that contradicted our findings, and have only reported instances where were unable to do so.

## Results

We present text from the FOIs in the sections that follow to explore firstly, whether ILSI is engaged in advocacy, and secondly if it is acting as a lobby group. Our view is to allow our medical and public health colleagues to explore and approach ILSI’s relationship with industry appropriately in the future when deciding whether to work with this body, receive its funding, and how to read studies and materials it produces.

### Is ILSI acting objectively or engaged in advocacy?

Notably, ILSI openly reports that it is industry funded [13, 14]. Suzanne Harris, however, attests that such funding does not lead to direct industry influence, presenting instead that ILSI is a “world leader”, acting to conduct “scientific inquiry relating to nutrition, food safety, toxicology, risk assessment and the environment” using a “tripartite operating model”, referring to input from industry, government and academia [8]. She suggests that this model is not only intended for “producing broadly informed scientific output”, but also at ensuring that “ILSI’s work is balanced and directed toward the public good …” [8]. Critically, then, we looked for instances that indicated either that ILSI promotes the public interest, or whether it sought to be selective in what it presented and promoted in ways that might support its industry members.

We found emails between Alex Malaspina, former senior vice-president at Coke and founder of, and long-time president of, ILSI, and Harris, amongst others, which suggest that ILSI protects industry from being affected by disadvantageous policy and laws. In response to new US dietary guidelines released that were unfavourable to industry, Malaspina writes:

*Dear Friends, These guidelines are a real disaster! They could eventually affect us significantly in many ways; Soft drink taxations, modified school luncheon programs ,a strong educational effort to educate children and adults to significanty limit their sugar intake,, curtail advertising of sugary foods and beverages and eventually a great pressure from CDC and other Agencies to force industry to start deducing drastically the sugar we add to processed foods and beverages, Also we have to expect that many nations will follow the US guidelines. We have to consider how to become ready to mount a strong defence. Warm regards. Alex* [Typographic errors his own] [15].

The inclusion of Harris among a large number of recipients may suggest that ILSI is perceived as having a role in countering public health nutrition policies that are unfavourable to producers of food and beverages with sugar added.

Other emails regarding ILSI’s research on the artificial sweetener saccharine likewise support this perception. Malaspina’s emails to Dr. James Emerson, a former Coke employee, [16] who sat on the ILSI Saccharin Technical Committee, [17] state:

*Dear Jim Emerson: Please note the extra praise for you from [former ILSI President] Sam Cohen. You and he were the architects to plan and execute the studies showing saccharine is not a carcinogen and all governments which had banned it reversed their position. Quite an achievement. Congratulations to both of you again* [18].

The email questions whether ILSI is engaged objectively, instead promoting industry positions to policy makers.

Critically, the FOI responses suggest that ILSI operates strategically with other industry-funded entities. Thus, Malaspina detailed the close relationship between ILSI and IFIC, which is a science communication charity, stating:

*… IFIC is kind of a sister entity to ILSI. ILSI generates the scientific facts and IFIC communicates them to the media and public* [19].

In an email chain between Malaspina and Michael Ernest Knowles, former vice-president of Global Scientific and Regulatory Affairs at Coca-Cola and former president of ILSI who remained a trustee of ILSI through 2016 and of the ILSI Research Foundation through 2017, both ILSI and IFIC are discussed. Malaspina details how “IFIC is coming through for our industry” and then proceeds to engage in a back-and-forth exchange with Knowles over strategies to “use external organizations” for product defence [20]. Knowles also notes the need for those in the sister entities to leverage positions within scientific societies to promote relevant work:

*we all belong to one or more of these and we should have leadership roles in the key ones and push for individual issues to be addressed by public conferences/ workshops in the manner of ILSI above* [20].

Overall, the emails suggest that both ILSI and IFIC act to counter unfavourable policies and positions, while promoting industry-favourable science, including to the media.

Concerningly, the FOIs showed instances of sanctions handed out by ILSI to its regional actors where they failed promote industry-favourable messaging. Malaspina details:

*… about the mess ILSI Mexico is in because they sponsored in September a sweeteners conference when the subject of soft drinks taxation was discussed. ILSI is now suspending ILSI Mexico, until they correct their ways. A real mess. … I have suggested to Suzie that to have ILSI Mexico save face, ILSI Mexico should join ILSI NA … . All of what has been happening is very, very sad for me, and I hope we have now reached Bottom [SIC] and eventually we will recover as Coke and ILSI are concerned* [21].

This suggests that ILSI’s constituent bodies face pressure to promote agreed positions by corporate members.

Taken together, these FOI emails suggest Mars Inc. was correct that ILSI engages in advocacy, and therefore we are left asking, does ILSI take this further by engaging in more substantive lobbying activity?

### Is ILSI lobbying?

While lobbying is understood in different ways in different jurisdictions, we understand it as where a group, organization, association or person attempts to influence governmental action (including executive, legislative, judicial and regulatory action) to achieve an outcome more favourable to their agenda. While the European Commission prefers the term “interest representation” over lobbying, capturing a broader set of activities designed to influence policy makers and decision-making processes, [22] other jurisdictions, such as the U.S.A. often employ a narrower concept focused on direct activities. Thus, U.S. legislation regarding defines it as “lobbying contacts and efforts in support of such contacts, including preparation and planning activities, research and other background work that is intended, at the time it is performed, for use in contacts, and coordination with the lobbying activities of others”, with lobbying contact meaning “any oral or written communication (including an electronic communication) to a covered executive branch official or a covered legislative branch official” [23]. When understood narrowly, this involves direct interaction to exert influence, including personal meetings and persuasion, while wider conceptions also consider indirect activities such as getting constituents to write letters, convening meetings and experts to exert influence, and creating a climate more favourable to meeting an objective, as included in lobbying. We sought to determine whether ILSI participates in either or both forms of this activity.

The FOI emails suggest ILSI promotes its agenda with national and international bodies to influence policy and law. The emails evidence on-going meetings with notable persons, and continuing concern about ILSI’s relationship with international bodies like the WHO, discussing how these bodies can be used to promote pro-industry positions. ILSI’s network is wide ranging. In emails between Malaspina and Barbara Bowman, then director of the US CDC’s Division for Heart Disease and Stroke Prevention, he states:

Dear Barbara

*You gave me some very good leads. I like the one especially about having Mr. Bill Gates help. Our Chairman knows him well. I will explore this idea with Clyde [Tuggle, Coke senior vice president]. We would want WHO to start working with ILSI again, with the GEBN and with the food industry in general to resolve issues of food safety and nutrition and for WHO to not only consider sugary foods as the only cause of obesity but to consider also the life style changes that have been occurring throughout the Universe.*

*Since WHO, as you stated has been helped by the pharmaceutical industry to combat HIV/AIDS, why not work closely with the food industry to combat obesity. The Food industry is very willing to come to the table.*

…

Warmest personal regards. Alex

Not only do we see that Bowman herself maintains a relationship with Malaspina and ILSI, but also is seen as a useful advisor regarding how to get ILSI into the WHO to push industry positions at the international level.

Emails between Malaspina and University of Washington Professor Adam Drewnowski support this role of ILSI with the WHO. Drewnowski explains how to develop ILSI’s influence with the WHO to further industry positions:

*Hello Alex - you are absolutely right about the need to start a dialogue with Margaret Chen [sic]. When she came to the Pacific Health Summit in Seattle some years ago, she said that she was ready to be "at the table - but not in bed - with industry" (her own phrase). Since then, her position has hardened considerably. We should remind her of her own phrase and get her to the table.*

*Now, strategically, we ought to start with some issue where ILSI and WHO are in agreement. What would that be? What are your thoughts?*

With best regards Adam

Follow-up emails Malaspina directed to Dr. Junshi Chen of ILSI China and China’s Centre for Disease Control, expand on this point:

*Dear Junshi: This is a great response by Adam on my email on WHO. Since you know WHO so well I thought you may have a suggestion as to how we can best handle this situation? Who do you recommend could be the person or organization to visit with her and discuss the science.? Warmest regards. Alex*

What becomes clear from the emails and forwards is that ILSI is seen as central to pushing pro-industry content to international organisations with a view to more industry-supported approaches that uncouple sugary foods and obesity.

The role of ILSI in defending industry from questions about the health consequences of its products and ingredients is seen in emails sent by Dr. James Hill, then director of the Center for Human Nutrition at the University of Colorado Health Sciences Center. In an email chain discussing public criticisms of scientists, societies and policy-makers working with industry, Hill states that:

*Frankly, we need the food industry to step up to provide more resources both to ILSI and GEBN. When things like this happen, individual companies tend to want to keep their head down. If they do this, our opponents will win and we will all lose.*

*I do not think we can overestimate the importance of dealing aggressively with this issue.*

*Jim* [24]

This makes clear the proactive role that ILSI is viewed as playing in addressing unfavourable actions, policies, and decisions. Additionally, it demonstrates that ILSI has academic affiliates who identify as being on the side of the industry.

Sometimes ILSI’s influence is even deeper, with individuals serving in ILSI posts while working for national and international bodies. For example, Junshi Chen and Wenhua Zhao served as Director and Deputy Director, respectively, of ILSI Focal Point China at the same time as working at the Chinese CDC [25]. Professor Alan Boobis simultaneously served as chair of ILSI’s Board of Trustees while he chaired the UN’s Joint FAO/WHO Meeting on Pesticide Residues panel on glyphosate, which was of great interest to ILSI major donors Monsanto and CropLife International [26]. The final meeting report included no conflict of interest statements, even though, as we demonstrate below, the WHO had, by the time of meeting ended, official relations with ILSI. It is clear that ILSI connected individuals continued to play a role in the WHO’s decision making, as well as holding a strategic position in relation to influencing national actors, like the CDC.

However, the FOIs indicate that the on-going relationships, both formal and informal, with international organisations including the WHO and Food and Agriculture Organization of the United Nations (hereafter FAO), as well as with the European Food Safety Authority (EFSA), are evolving and increasingly ILSI is being seen as a private actor. In 2017, ILSI was no longer recognized as in special relations with the WHO, and its links to EFSA were the subject of enquiry at European Parliament and new guidelines on transparency [22]. Many of our FOIs predate these changes, but reveal why increasing recognition of ILSI’s activities on behalf of industry have led it to be increasingly viewed as a private entity with conflicts of interest.

Notably, the FOIs make clear ILSI was previously deployed to influence the WHO Director General Margaret Chan, who had promoted policies and research contrary to the interests of those selling sugar-sweetened products, with Malaspina stating:

*We must find a way of some one such as a famous scientist arrange to pay her a visit. Jim Hill or some one of similar stature or a US Government scientist. As the President of ILSI I had a special and productive luncheon with the former DG, Dr Nakajime [SIC] in 1995 at his private dining room in the WHO Geneva Headquarters to tell him about ILSI and how the two organisations could work with each other. In 1999 I visited the new DG Mrs Brutland in Geneva, when I invited her, on behalf of the World Economic Forum, to come to the Davos meeting of 1999, and be the Keynote Speaker at the Food Governors special dinner … By the way, the future Coke President, Mr Neville Isdell attended that dinner with me. In summary I am suggesting that collectively we must find a way to start a dialogue with Dr Chen [SIC]. If not, she will continue to blast us with significant negative consequences on a global basis. This threat to our business is serious.* [Typographic errors his own] [27]

In subsequent emails, Malaspina asks Bowman:

*Any ideas how we can have a conversation with WHO? Now, they do not want to work with industry. Who finds all the new drugs. Not WHO, but industry. She is influenced by the Chinese Govt and is against US. Something Must be done [sic].*

Bowman then proceeds to provide advice about whom to approach to influence the WHO on sugar and beverage policy matters, and to promote ILSI’s central role in influencing the policy arena; an email chain that when revealed garnered international press, and resulted in Bowman’s subsequent resignation from the CDC [28].

Following this 2015 exchange, Harris asked the ILSI board of trustees to consider an amendment to the membership section of the ILSI bylaws as a way of addressing a concern recently raised by the WHO regarding one of ILSI’s members being linked to tobacco [29]. In the minutes, it is noted that the WHO proposes to class ILSI as a “private sector” entity, a classification which is wholly unacceptable to ILSI.

In a 2015 email, Eric Hentges detailed to ILSI members, academic collaborators and others that:

*… in late January 2015, the World Health Organization’s Executive Board decided to discontinue official relations with the International Life Sciences Institute (ILSI)* [30].

This email chain indicates that, despite efforts to amend its bylaws to maintain official relations with the WHO, the WHO chose to remove ILSI from its lists, thereby removing special status.

## Discussion

Our analysis of the FOI documents does not portray ILSI as a neutral, independent scientific charity acting to improve the public’s health and well-being around the world. We identified overt attempts by ILSI to influence individuals, positions, and policy, both at national and international levels, alongside clear statements that ILSI’s corporate members deploy it as a tool to thwart policies or leaders who are hostile to their interests. Policy makers around the world, and our medical, public health, and nutrition colleagues, should be mindful of this industry influence when approaching studies funded by ILSI, which regularly appear in leading journals, and when receiving ILSI perspectives on the evidence-base to support policy making.

Before interpreting the implications of our findings for policy and future research on the commercial determinants of health, we must first acknowledge its important limitations. First, as with all FOI-based research, there is potential for biases in our interpretations. To mitigate this, we have quoted all text directly and in context, allowing the documents to speak for themselves. Further, we make all material cited available in accompanying web links so that readers can interrogate our interpretations of source material and conduct research with the batches themselves. We sought to actively look for instances that refuted our interpretations and were unable to do so in our document set.

For policy, our data suggests it is necessary to question whether ILSI (along with related bodies like IFIC and some of those listed in the Introduction) should be, as it is currently, regarded as a charitable actor. The WHO has asked whether ILSI should be regarded as a private sector entity. ILSI recognises that such a change in categorisation would greatly circumscribe its role in policymaking, especially as pertains to public health, and lead those in public health to approach its work with appropriate caution, acknowledging industry influence.

Given our evidence, we contend that ILSI should be regarded as a lobby group and that academics and researchers, policy makers, the media, and the public should view ILSI’s research as promoting the interests of the food, beverage, supplement and agrichemical industries, while its actions promote its members interests and counter healthy public policies.

## Conclusions

While ILSI purports to be working for health and wellbeing of populations internationally, we identified attempts by ILSI to influence individuals, professional guidance, and policy, both locally and internationally, while we saw evidence that its corporate members deploy it as a tool to promote their interests globally. As such, policy makers, international bodies, and the medical and research communities, should approach ILSI’s work with caution, viewing it as industry funded and influenced. Regulators should also consider ILSI’s status as a lobby group in Europe, the Americas, and beyond. Our analysis of ILSI serves as a caution to those involved in global health governance to be wary of putatively independent research groups, and to practice due diligence before relying upon their funded studies and/or engaging in relationship with such groups.
