# Correction to: Are industry-funded charities promoting “advocacy-led studies” or “evidence-based science”?: a case study of the International Life Sciences Institute

**DOI:** 10.1186/s12992-019-0512-8

**Published:** 2019-11-06

**Authors:** Sarah Steele, Gary Ruskin, Lejla Sarcevic, Martin McKee, David Stuckler

**Affiliations:** 1 Department of Politics and International Studies, University of Cambridge, Cambridge, UK; 2 Jesus College, Jesus Lane, Cambridge, CB58BL UK; 3 U.S. Right to Know, Cambridge, USA; 4 Department of Public Health and Policy, London School of Hygiene & Tropical Medicine, London, UK; 5 Dondena Research Centre and Department of Policy Analysis and Public Management, University of Bocconi, Milan, Italy

**Correction to: Glob Health (2019) 15:36**

**https://doi.org/10.1186/s12992-019-0478-6**

Since the publication of this article [1], the journal and the authors have received further context about the position of ILSI on the issue with the ILSI Mexico branch.

ILSI have provided redacted minutes, four pages in length, from an extraordinary conference call dated 2 November 2015 with the ILSI Board of Trustees Executive Committee. This document, unavailable publicly, describes the actions taken by ILSI Mexico as amounting to lobbying or advocacy, and suggests the courses of action open to ILSI’s Executive Committee related to this violation of ILSI’s Code of Ethics/Standards of Conduct. In light of these minutes, we believe that the correspondences we cite related to ILSI Mexico should be reinterpreted, and the authors wish to correct the record.

In these minutes, ILSI offers to suspend ILSI Mexico’s charter for a fixed period of time, rather than removing the organisation’s charter, until the organisation demonstrates an understanding of ILSI’s policies and willingness to abide by them. ILSI proposes exercising significant oversight of the branch, a change in personnel, and the submission of an annual plan. ILSI Mexico was subsequently reinstated in September 2016.
